# Trends and Geographic Variabilities in Benzodiazepines Prescription in Primary Care to Older Adults: A 3-Year Population-Based Ecological Study in Portugal

**DOI:** 10.3390/healthcare10071342

**Published:** 2022-07-19

**Authors:** Ana Bárbara Tavares, Ana Isabel Placido, Daniela Almeida Rodrigues, Manuel Morgado, Adolfo Figueiras, Maria Teresa Herdeiro, Fátima Roque

**Affiliations:** 1 Research Unit for Inland Development, Polytechnic of Guarda (UDI-IPG), 6300-559 Guarda, Portugal; barbaratavares@ipg.pt (A.B.T.); danielaalmeidar@ipg.pt (D.A.R.); mmorgado@ipg.pt (M.M.); 2 Health Sciences Research Centre, University of Beira Interior (CICS-UBI), 6200-506 Covilha, Portugal; 3 Pharmaceutical Services of University Hospital Centre of Cova da Beira, Quinta do Alvito, 6200-251 Covilha, Portugal; 4 Consortium for Biomedical Research in Epidemiology and Public Health (CIBERESP), 28001 Madrid, Spain; adolfo.figueiras@usc.es; 5 Health Research Institute of Santiago de Compostela (IDIS), 15706 Santiago de Compostela, Spain; 6 Institute of Biomedicine, Department of Medical Sciences, University of Aveiro, 3810-193 Aveiro, Portugal; teresaherdeiro@ua.pt

**Keywords:** benzodiazepines, older patients, prescription trends, Portugal

## Abstract

(1) Background: According to the World Health Organization (WHO), benzodiazepines (BZD) are considered essential medicines for the treatment of several mental disorders in older adults over 65 years old. However, the long-term use of BZD could present a harmful impact on this population, leading to cognitive deficits, drug dependence, falls, and fractures. This study aims to analyze trends of BZD prescription to Portuguese older adults in the primary care setting, and to analyze the change in the prescription rate of BZD over time, assessing the geographical variability in mainland Portugal. (2) Methods: A nationwide, retrospective ecological study was performed between January 2019 and December 2021 for BZD prescribing data reported in a national public database for all persons aged 65 and older in mainland Portugal (about 2.4 million). Trends of BZD by defined daily doses (DDD) and per 1000 older adults’ inhabitants per day (DID) were analyzed. (3) Results: A total of 19 BZD were included in this study and more than 1 million BZD prescriptions were recorded in each year of this study period. BZD prescriptions were three times higher in females than in males. Alprazolam, lorazepam, diazepam, ethyl loflazepate, and bromazepam were the most prescribed BZD over the years, presenting the higher DDD and DID values. (4) Conclusions: Despite the DID value growth of several BZD, Portugal is now showing stable BZD prescriptions in older adults, between the years 2019 to 2021. More studies are needed to access if these results are a consequence of successful health programs or just a consequence of the pandemic context that we are facing, which limited older adults’ clinical appointments.

## 1. Introduction

Benzodiazepines (BZD) are commonly prescribed medicines for the treatment and management of anxiety, depression, and insomnia in older adults, due to their anxiolytic, sedative and hypnotic, and muscle relaxant properties [1,2,3]. This pharmacological group is considered extremely effective in short-term treatment, typically between 8–12 weeks for anxiety, and no more than 4 weeks for insomnia, losing effectiveness when used beyond the recommended period [2,4]. The prolonged use of BZD in older adults frequently leads to drug dependence and tolerance to their sedative and anxiolytic properties, resulting in an increased sensitivity towards their harmful side effects, such as cognitive impairment, falls, and fractures [2,5,6]. As a matter of fact, the harm associated with the long-term use outweigh the possible benefits of BZD in older adults, which is why several BZD are considered potentially inappropriate medication (PIM) for this population [7,8,9,10]. 

According to the report of the Organization for Economic Co-operation and Development (OECD), in 2017, Portugal was in the top three of the 12 European Union countries with the highest defined daily dose (DDD) per 1000 population per day (DID) value for BZD chronic use (65 DID) [11]. For that reason, over the years, certain measures have been taken to encourage BZD deprescription in Portugal. The National Program for Mental Health, published in 2017, proposed to reverse the trend in BZD prescription by stabilizing the prescription of medications for the treatment of anxiety until 2020 [12]. In addition, the Central Administration of the Health System (ACSS—Administração Central do Sistema de Saúde) created a protocol on the chronic use of BZD, aiming to evaluate interventions to discontinue their use in primary health care [13]. Portugal has one of the most complete National Health Systems (NHS—Serviço Nacional de Saúde), which is structured as a nationwide tax-based public system that allows integration and complementarity between different levels of care, where every Portuguese person is inserted and properly attributed to a general practitioner [14,15,16]. Furthermore, it is precisely the public health system, in particular, primary care health, that is responsible for most of the BZD prescriptions in Portugal [4,17].

Despite the countless guidelines advising BZD deprescription in older adults and the fragility presented by this population, epidemiological data reveals that there is a misuse of BZD among older adults in the primary care setting [17,18]. Considering that primary care physicians are mostly the leading prescribers for older adults, the main aim of this study is to analyze trends of BZD prescription to older adults in primary care, and to analyze the change in the prescription rate of BZD over time, assessing the geographical variability between different regions of mainland Portugal.

## 2. Materials and Methods

### 2.1. Study Design and Study Population

A retrospective, ecological study was conducted using Portugal’s official System of Information and Monitoring of the Portuguese National Health System (SIM@SNS) public access online platform [19], an anonymized database created by the Shared Services of the Health Ministry (Serviços Partilhados do Ministério da Saúde—SPMS), to provide aggregated data to all entities, stratified according to sex, age, and Portugal Region. Through this database, data on BZD prescription in primary health care was collected for the population aged 65 years old and over, in mainland Portugal, from January 2019 to December 2021. 

The Reporting of Studies Conducted Using Observational Routinely Collected Health Data (RECORD) for Pharmacoepidemiology (RECORD-PE) standard guidelines were followed when applicable, as per recommended practice (Supplementary Data).

### 2.2. Benzodiazepines Studied

At the time of this study, and according to the ATC Index, 39 drugs are classified as benzodiazepines. However, for the purposes of this study, a total of 20 BZD were excluded, given the following exclusion criteria: (a) BZD without marketing authorization (MA) or had lost MA in Portugal during the study period (n = 18) [20]; (b) BZD without DDD (n = 1) [20]; and (c) BZD exclusively used in a hospital setting (n = 1) [20] (Table S1 in Supplementary Material). The remaining 19 BZD were included in this study, being classified according to the ATC Code. Diazepam (N05BA01), chlordiazepoxide (N05BA02), oxazepam (N05BA04), potassium clorazepate (N05BA05), lorazepam (N05BA06), bromazepam (N05BA08), clobazam (N05BA09), prazepam (N05BA11), alprazolam (N05BA12), ethyl loflazepate (N05BA18), cloxazolam (N05BA22), and mexazolam (N05BA25) are classified as anxiolytics (N05B group) and represent 63.16% of the total BZD enrolled in this study. Flurazepam (N05CD01), estazolam (N05CD04), triazolam (N05CD05), temazepam (N05CD07), midazolam (N05CD08), brotizolam (N05CD09), and loprazolam (N05CD11) are responsible for the remaining percentage of BZD (36.8%) and are classified as hypnotic and sedatives (N05CD group). 

### 2.3. Data Source

The online database was accessed in March 2022 and the BZD-DDD prescriptions data were fully extracted. The following information was also extracted: gender (female/male), age group (65–74 and ≥75 years old), and Portugal’s Regional Health Administrations (Administração Regional de Saúde—ARS), which are divided into five regions: North (ARSN), Centro (ARSC), Lisbon and Tagus Valley (ARSLVT), Alentejo (ARSALE), and Algarve (ARSALG) (Figure S2—Supplementary Material).

### 2.4. Statistical Analysis

The BZD prescription data were analyzed from four different perspectives: (a) Total BZD prescription share (%)—calculated as the percentage of BZD-DDD in the total of DDD medicines prescribed in older adults, given the same time period, ARS, gender, and age group; (b) Most prescribed BZD (%)—which relates the prescription of a specific BZD-DDD with the total of BZD-DDD prescription during the same period, ARS, gender, and age group; (c) DDD per 1000 older adults per day (DID)—calculated based on the Statistics Portugal data for the older population, according to each region in Portugal [21]; and (d) change rate (%), calculated as the difference between DID value in 2021 and in 2019 divided by the DID value in 2019. The cumulative DID value was determined based on the average number of older patients throughout the 3 years of the study. Every indicator was calculated based on the WHO ATC/DDD Toolkit [22].

## 3. Results

### 3.1. Study Population 

This study comprises a population of 2.4 million older people aged at least 65 years old—where about 58% are females, randomly distributed by Portugal’s Regional Health Administrations (ARS—Administração Regional de Saúde) of North (ARSN), Center (ARSC), Lisbon and Tagus Valley (ARSLVT), Alentejo (ARSALE), and Algarve (ARSALG) (Figure S2 in Supplementary Material) [21]. Furthermore, the major metropolitan areas of Portugal, ARSLVT and ARSN, are accountable for a significant percentage of the older population—the two combined have more than 50% of older people, when compared to the remaining ARS. Conversely, ARSALG shows the lowest values for the older population in Portugal, with a percentage no greater than 4.5% (Tables S2–S4 in Supplementary Material). 

### 3.2. Total Benzodiazepine Prescription Share (%)

**Figure 1:**
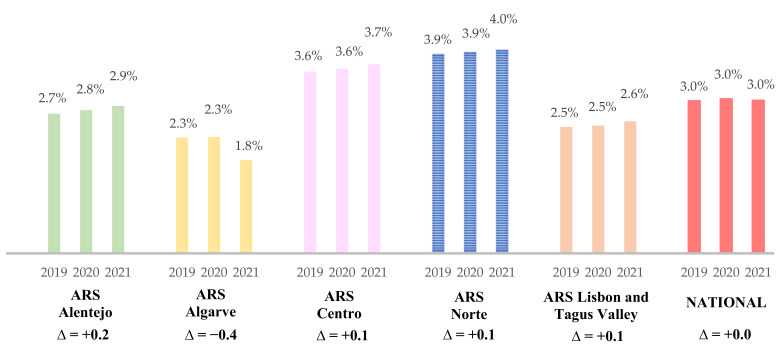
Total benzodiazepine prescription share (%) in older adults at a National Level and by ARS, from 2019 to 2021. Variance level (∆) presented. ARS—Portugal’s Regional Health Administrations (ARS—Administração Regional de Saúde).

Overall, a total of 1.5, 1.6, and 1.7 million DDD of BZD were prescribed to older adults in 2019, 2020, and 2021, respectively. The BZD-DDD/total medicines-DDD prescription (total benzodiazepine prescription share %) in older adults is described in Figure 1, according to each ARS and between the years 2019 and 2021. The lowest rate (1.8%) was obtained in ARSALG, whereas the North region reports the highest, with 4.0%, both in the year 2021. It is notable that there was a slight increase in BZD prescriptions to older adults from 2019 to 2021 in all ARS from mainland Portugal, except for the South region, which reports a negative variation of −0.4%. Concerning BZD prescription by ARS, a positive variance of +0.2%, +0.1%, and +0.1% was observed in the ARSALE, ARSC, and ARSN, respectively. However, it is shown that Portugal’s tendency in BZD prescription to older adults leans to a stabilization between 2019 and 2021.

#### 3.2.1. Benzodiazepine Prescription by Gender

At a national level, and concerning gender prescription, it was observed that BZD prescription is 2.7 times higher in older females than in older males (>65 years), as represented in Table 1 (Table S5 in Supplementary Material). Taking into consideration all the 19 BZD included in this study, the female/male ratio (F/M) reveals a value superior to two in every ARS, ARSALE being the one with the highest relation, with female patients having almost three times more BZD prescriptions than male patients (3.0 precisely), whereas the North region shows the lower value, with 2.4.

#### 3.2.2. Analysis of Most Prescribed Benzodiazepines Drugs

Taking into consideration the time of this study, 92.6% of the total BZD prescriptions are for anxiolytics BZD (N05BA group), whereas only 7.4% of BZD prescriptions are for hypnotic and sedative BZD (N05CD group) (Table S6, Supplementary Material). Due to its high prescription, a more detailed analysis was conducted on the N05BA group, particularly on the five most prescribed: alprazolam, lorazepam, diazepam, ethyl loflazepate, and bromazepam, since these five BZD are responsible for more than 85% of all the BZD-DDD prescriptions (Table S7, Supplementary Material). Furthermore, this top five was found to be the same in every ARS of mainland Portugal.

**Figure 2:**
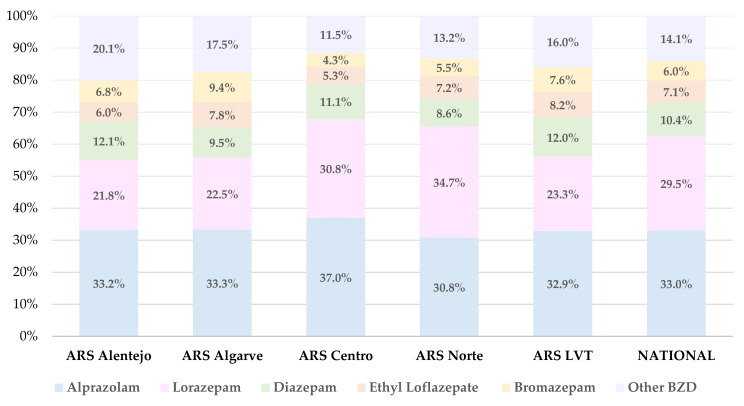
Percentage of alprazolam, diazepam, ethyl loflazepate, bromazepam, lorazepam, and other BZD, giving the total number of prescribed BZD, according to each ARS and at a national level, between 2019 and 2021.

Figure 2 represents an analysis of the percentage of each BZD of the top five included in the study, giving the total number of prescribed BZD in each ARS of mainland Portugal between the years of 2019 to 2021. At a national level, alprazolam holds the highest frequency of BZD prescription in mainland Portugal (33.0%), followed by lorazepam (29.5%). Ethyl loflazepate and bromazepam are the least prescribed BZD in Portugal, with a frequency of 7.1% and 6.0%, respectively. However, the remaining 14 BZD included in this study are accountable for only 14.1% of the nationwide BZD-DDD prescriptions, revealing a minor value in terms of BZD-prescription, in comparison to the BZD from the top five. 

Alprazolam was the most prescribed BZD in all the ARS of mainland Portugal, except for ARSN, in which the most prescribed BZD was lorazepam (30.8% vs. 34.7%, respectively). Alprazolam achieves its higher frequency in ARSC with 37.0%, whereas ARSALE is where diazepam shows its top value (12.1%). Ethyl loflazepate was most prescribed in ARSLVT (8.2%), whereas ARSALG holds the highest prescription for bromazepam, with 9.4%. The lowest rate of BZD prescription was detected for bromazepam, with only 4.3% in ARSC. For the BZD excluded from the top five, they achieve their highest value in ARSALE, with 20.1%, whereas ARSC holds the lowest value, with 11.5%. 

### 3.3. Trends and Change in the Prescription Rate of Benzodiazepines

**Figure 3:**
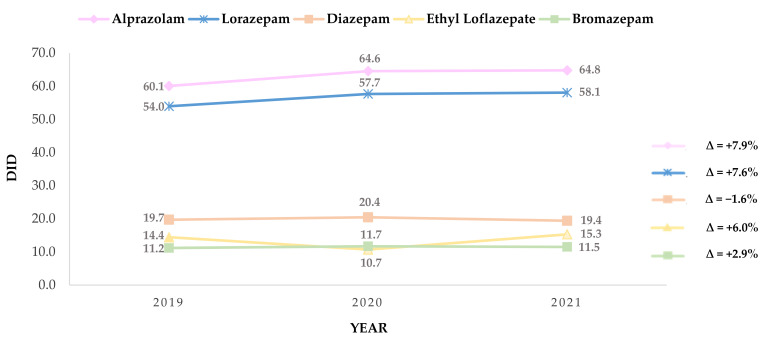
Prescription and variance (%) over the years of alprazolam, lorazepam, diazepam, ethyl loflazepate, and bromazepam, expressed in DDD per 1000 older adult inhabitants per day (DID).

Prescription in older adults over the last 3 years for alprazolam, lorazepam, diazepam, ethyl loflazepate, and bromazepam, at a national level, is represented in DID in Figure 3, as well as the variance in DID value (%) over the same time period. 

During the study period, the DID in older adults for alprazolam increased from 60.1 in 2019 to 64.9 in 2021, establishing the major continuous growth of the top five BZD (+7.9%). Accordingly, lorazepam reveals a change rate of +7.6% over the years (54.0 to 58.1, in 2019 and 2021, respectively). Regarding the remaining top five BZD, minor results are presented, diazepam being the BZD that displays the greater decrease (−1.6%) in DID, from 19.7 in 2019 to 19.4 in 2021. Ethyl Loflazepate presents a decline in DID value between 2019 and 2020, from 14.4 to 10.7, respectively, although this value rises rapidly in 2021 (15.3), proving a growth of +6.0%. Concerning bromazepam, the results are mostly persistent throughout the years, presenting 11.2 DID in 2019, 11.7 in 2021, and 11.5 in 2021, resulting in a change rate of only +2.9.

In terms of gender and age group prescription, detailed data analysis revealed severe differences in DID prescription values between female and male older adults and their age groups (Table S8—Supplementary Material). Female predominance is clear, since this gender holds the highest values of prescription for every BZD of the top five in every ARS of mainland Portugal between the years 2019 to 2021, in comparison to the male gender. Regarding the age group, older adults over 75 years old tend to show higher DID values for BZD prescriptions, despite some decreases being recorded in a few BZD, from 2019 to 2020, for this age group. 

Over the last three years, ARSN and ARSC are accountable for the highest DID in older adults of every BZD of the top five. The highest DID was obtained in 2020, in ARSN, where lorazepam (113.3) was the most prescribed for females over 75 years. The lowest DID occurred in male older adults, in ARSALG, where ethyl loflazepate achieves the value of only 3.6 for males over 75 years. Ethyl loflazepate is also responsible for the lowest DID in the female gender, with 7.5 in 2020 in ARSALE. Conversely, lorazepam was the most prescribed BZD in ARSN in 2020 for the male gender, with 67.0.

### 3.4. Geographic Variation in DID Benzodiazepine Prescription to Older Adults

Since the most prescribed BZD are the same for all the ARS of mainland Portugal, Table 2 illustrates the DID change rate of the top five BZD, in each ARS, stratified by year. Between 2019 and 2021, ARSN was the only ARS that registered an increase in all the five BZD, whereas for ARSALG, only decreases were recorded.

It was in the North that the alprazolam DID value increased the most (14.1%), closely followed by ARSLVT (11.9%). ARSN was also accountable for the highest DID value for loprazolam (12.8%) and diazepam (7.2%). Furthermore, both alprazolam and lorazepam revealed their maximum value decrease in ARSC (−4.4% and −2.2%, respectively). Diazepam displays its major value reduction in the Center region (−11.6%), although ARSALG (−10.9%) and ARSALE (−9.3%) also show high and similar decreases in DID value for this BZD. Regarding the DID value of ethyl loflazepate, ARSLVT shows a considerable increase (14.2%) and ARSALG shows a decrease (−0.8%). Concerning bromazepam, only ARSN and ARSLVT reveal a DID increase, both with 6.8, whereas ARSC shows the highest decreased value for this BZD (−7.2%), followed by ARSALG, with −4.0%, and ARSALE, with −1.2%.

Geographic differences from the five ARS of mainland Portugal, relating prescription in DID value for the top five BZD, are also presented in Table 2. However, a heterogeneity can be seen in the geographic distribution for each BZD (Figure S1A–E—Supplementary Material). Concerning alprazolam, ARSC was accountable for the superior level of prescriptions, whereas for lorazepam, the North region registered the highest DID value over the years. For diazepam, only ARSC distinguishes itself from the other ARS. Ethyl loflazepate was mostly prescribed over the last years in ARSN, followed by ARSLVT. Bromazepam revealed a more similar prescription between each ARS, with ARSN, ARSLVT, and ARSALE being the main regions where the highest number of prescriptions were recorded. ARSALG was the ARS that verified the lowest prescriptions in every BZD of the top five, except for bromazepam, which reports this value for the Center region.

## 4. Discussion

This study shows a stable BZD prescription in Portugal, where no national increase was shown during the 3-year study period (2019–2021). Considering that these data overlap with the worldwide pandemic, when analyzing the impact of the COVID-19 on BZD prescription and consumption, a Portuguese study reported a slight increase in the prescription of anxiolytics, sedatives, and hypnotic drugs in older adults over 65 years, especially in females, through one year of the COVID-19 pandemic [23,24]. Other European countries share this reality, where an increase in prescriptions for these medicines was also described [25,26]. In that manner, the stable results achieved in our study could be related to the success of the implemented Health Programs that aimed to establish or even decrease BZD Prescription in Portugal [12,27]. 

According to the OECD in 2017, Portugal’s chronic consumption of BZD in DIDs was far over the European average (65 DID vs. 32 DID, respectively) [11]. Considering these results and given Portugal’s status as one of the highest consumers of BZD, in 2017, the Portuguese National Direction of Health (DGS—Direção Geral de Saúde) established new Goals of Health for 2020, a health-related program that includes mental health problems as one of the focuses of the program design, where the aim is to reverse the national tendency towards high BZD prescriptions, achieving future stability [27]. After the implementation of this program, the latest OECD Report (2021) unveils a significant decrease in Portugal’s chronic BZD use in 2019, with a DID value of 37.2, indicating that the settled goals were beyond achieved [28]. Nevertheless, our results, which covers the years 2019 to 2021, show stability in terms of BZD-DDD prescription instead of a continued decrease, which could be related to the effect of the COVID-19 pandemic in our country. COVID-19 had a harmful impact in 2020 and 2021, adversely affecting the population’s mental health at a worldwide level, and consequently leading to a higher level of prescription and consumption of benzodiazepines and antidepressant drugs [11,23,26,29,30]. Despite the unfavorable circumstances for mental health created by the pandemic, which put Portugal as the 3rd heaviest consumer country of BZD worldwide in 2020 (according to the International Narcotic Control Board (INCB) 2021 report), Portugal’s rates in BZD prescriptions achieved a stable value in 2021 according to our data, preventing what could have been an escalated increase due to the pandemic, and revealing the success of the new Goals for Health over the years [31]. These results could also be associated with the decrease in hospital visits and/or primary care clinical appointments during the pandemic period, due to lockdown, which averted a possible increase in BZD prescriptions [23]. 

The majority of BZD prescriptions (92.6%) are anxiolytics (N05B), whereas only a small percentage of 7.4% are hypnotic or sedative BZD (N05CD), which reveals a prescribing preference for anxiolytic BZD rather than for the sedative or hypnotic ones [17,32,33,34]. This predisposition may possibly be related to the strong sedative effect caused by N05CD-BZD, which ultimately leads to a prescribing preference towards slightly less soporific BZD, such as anxiolytics, assuring a milder effect in the central nervous system (CNS) [35]. Furthermore, the predominance of anxiety, depression, stress, loneliness, and mild insomnia—mental health issues commonly and advisedly treated with anxiolytics—in Portuguese older adults, could also contribute to this prescribing tendency [4,36,37,38,39,40]. Nevertheless, this direct association cannot be established because of a lack of data in our database, in terms of clinical indication for prescription, although anxiety has been reported as one of the main diseases affecting older adults, supporting our hypothesis [39]. In fact, the INCB has formerly reported this startling tendency in Portugal, which mainly relies on the prevalence of mental disorders in the Portuguese population [31,33,41,42]. Accordingly, a nationwide study from 2018, reporting the World Mental Health Surveys (WMHS) in Europe, revealed a 12-month prevalence of mental disorders of 21% for the Portuguese population, which was only surpassed by Northern Ireland [38,43]. 

From the 19 BZD included in this study, our results indicate that, from 2019 to 2021, alprazolam, lorazepam, diazepam, ethyl loflazepate, and bromazepam were the most prescribed. According to a study from the National Authority of Medicines and Health Products, I.P. (INFARMED—Autoridade Nacional do Medicamento e Produtos de Saúde, I.P) on Benzodiazepine’s Utilization, and the DGS report “Portugal: Mental Health in Numbers—2015” (“Portugal: Saúde Mental em Números—2015”), alprazolam was already the most prescribed BZD in Portugal in 2015, followed by lorazepam, diazepam, and ethyl loflazepate [17,33,40]. Every ARS of mainland Portugal shows a higher prescription for alprazolam, except for ARSN, where lorazepam was the most prescribed (30.8% vs. 34.7%, respectively) according to our data. In terms of DID value, in the past three years, our data suggest a DID increase for all the selected top five BZD, except for diazepam, the only BZD that reports a decrease of −1.6% between 2019 and 2021. Alprazolam establishes major growth, with an increase of +7.9% in DID value in the same years, closely followed by lorazepam, with +7.6%.

Despite Portugal being a small country in comparison with others in Europe, it is extremely heterogeneous in terms of population health literacy, important in the geographic analysis of the data [44]. Moreover, Portugal geography also reveals heterogeneity in terms of access to health services, general practitioner, and socioeconomic status, which ultimately affects the periodicity and number of clinical appointments, prescriptions, and patient follow-ups [45,46,47]. Due to the overburden of physicians, it has been very difficult to maintain the follow-up of patients according to the advised guidelines periods. On the other hand, the health literacy of the population and difficulties of deprescribing by physicians are also responsible for several drug-related problems, which result in short-term prescriptions to be extended over time [48,49]. During the study period and considering the total benzodiazepine prescription share (%), ARSALG unveils the maximum decrease in BZD-DDD prescription, with a variance of −0.4% over the last three years, whereas the remaining ARS all show a slight increase in terms of BZD-DDD prescriptions, although keeping the variance at a maximum level of only +0.2% for ARSALE. Despite the decrease in prescriptions of several BZD from the top five, heterogeneity in BZD-prescription is present in our geographical data. For ARSALG, a decrease in each BZD DID value was recorded, whereas for ARSC, despite the general decrease, ethyl loflazepate shows a +7.0% increase. Nevertheless, the North region only shows increased prescriptions in the studied period, revealing significant and alarming growth in the DID value for alprazolam (+14.1%) and lorazepam (+12.8%), which maintains the geographic tendency revealed in the 2016 INFARMED report about the use of BZD in Portugal [17]. 

All the benzodiazepines included in this study were highly prescribed to older females, in comparison to older males, in all the ARS of mainland Portugal, showing a twofold model of prescription for this gender. Portugal is reported as one of the European countries with the highest rate of chronic depression and prevalence of psychological distress symptoms, with these values prevalent in older females [11,50]. Moreover, women are more prone to be diagnosed with anxiety and show a biological tendency to be more susceptible to depression than males, which is aggravated by menopausal and postmenopausal symptoms [51,52]. Eurostat indicates that for chronic depression in Portugal, females show a rate of 26% (the highest in Europe), whereas the male gender reveals a percentage of only 10.7% for older adults over 65 years [50]. These results, which are in accordance with our data, reveal a predominance in terms of consumption and prescriptions for the female gender, showing the likelihood of women being the higher consumers of these medicines [17,51]. 

Regarding the age criteria of this study, the same was chosen based on the clear prevalence of BZD prescription in older adults [17,38,53]. The American Geriatrics Society (AGS) published an updated AGS Beers Criteria in 2019, in which they strongly advised to avoid the prescription of these medicines to older adults, since they show a high vulnerability to benzodiazepines, and their consumption is often associated with severe adverse reactions [9,40]. In fact, due to the pharmacokinetic and pharmacodynamic changes, comorbidities, and cognitive and functional aspects inherent to the aging process, this pharmacological group is considered a potentially inappropriate medication (PIM) in older adults, according to the main PIM criteria described in the literature: Beers criteria, EU (7)-PIM List, and the Screening Tool of Older Persons’ Prescriptions and Screening Tool to Alert to Right Treatment (STOPP/START) [7,8,54]. Generally, BZD are associated with an increased risk of cognitive impairment, delirium, falls, and fractures in older adults. Besides, due to their anticholinergic properties, BZD can cause orthostatic hypotension and strong sedation, which leads to high rates of dependence and risk of overdose in cases of low-dosage prescription [9,38,54,55]. Despite the advisements towards decreasing the prescription of BZD in the older population, and possibly due to an attempt to increase their quality of life, these drugs are still commonly prescribed for anxiety, sleep disorders, and depression among older adults [11,56]. In fact, a Portuguese study on perceptions and beliefs of health professionals reports that primary care physicians themselves state that deprescription in older adults is extremely difficult, since most of the medication is chronic and their absence could diminish the quality of life [48]. According to our data, the higher registered DID values are from older adults over 75 years, and as the INFARMED reports in 2016, the consumption of BZD tends to increase with age [2,17]. A further challenge takes place in terms of the period of treatment, since BZD are advisedly prescribed for a short period of time (between 4 to 12 weeks, depending on the cause of treatment), but their use frequently becomes chronic among older adults due to the prevalence of mental disorders in this population [4]. In Portugal, older adults aged > 65 years reveal a value of 65 DID in chronic consumption of BZD, which constitutes more than the double the consumption the OECD-EU average presents (32 DID) [11]. However, the withdrawal of this kind of medication may constitute a problem, since it largely affects the quality of life of an older adult. In consequence, and as shown by our data analysis, Portugal’s trends in BZD prescription in older adults tend to stagnate; despite the attempt to lower BZD prescription for older adults who are starting a new treatment, the older patients already on BZD tend to continue this course of treatment, assuring their quality of life [56]. Hence, these results may indicate a positive and successful outcome from the implementation of the new Goals of Health 2020, as is shown in our results through a national stagnation in Portugal’s BZD-DDD prescriptions for older adults, as recommended by DGS. 

The major strength of this study is the population, since all older adults over 65 years from mainland Portugal are included for analysis, despite their health status. Besides, since the NHS is entitled to all the Portuguese population, it is possible to obtain a representative sample of the Portuguese population in this age group. Nevertheless, despite the relevant data, this study presents some limitations. Firstly, the study only includes the BZD prescription data of outpatient care in the public sector, excluding possible BZD that could have been administered in hospital settings and/or in the private health sector. Furthermore, the data from System of Information and Monitoring of the Portuguese National Health System (Sistema de Informação e Monitorização do Sistema Nacional de Saúde—SIM@SNS) used in this study did not report the specific clinical indication for BZD prescription in this population in particular. 

## 5. Conclusions

The data obtained from this study underline Portugal’s stability in BZD prescription in older adults between the years 2019 and 2021. The prescription tendency of BZD was higher in older females for all the BZD included in the analysis. Alprazolam, lorazepam, diazepam, ethyl loflazepate, and bromazepam were the most prescribed BZD at a national level and in every ARS of mainland Portugal. 

## Figures and Tables

**Table 1:** Female and male ratio in terms of BZD-DDD prescriptions over the 3 years.

	Alentejo	Algarve	Norte	Centro	LVT	National
F/M	3.0	2.6	2.4	2.7	2.8	2.7

**Table 2:** DID change rate (∆ in %) of the top five benzodiazepines, in each ARS, stratified by year.

Benzodiazepine	2019 (DID)	2020 (DID)	2021 (DID)	∆ 2021–2019 (DID)	∆ 2021–2019 (%)
ARSN					
Alprazolam (N05BA12)	63.1	72.0	71.9	8.9	14.1%
Lorazepam (N05BA06)	71.6	80.9	80.8	9.2	12.8%
Diazepam (N05BA01)	18.1	20.2	19.4	1.3	7.2%
Ethyl loflazepate (N05BA18)	16.9	13.4	18.2	1.3	7.4%
Bromazepam (N05BA08)	11.6	12.8	12.4	0.8	6.8%
ARSC					
Alprazolam (N05BA12)	78.8	79.5	75.4	−3.4	−4.4%
Lorazepam (N05BA06)	65.1	65.9	63.6	−1.5	−2.2%
Diazepam (N05BA01)	24.6	24.2	21.7	−2.9	−11.6%
Ethyl loflazepate (N05BA18)	12.9	8.8	12.0	−0.9	7.0%
Bromazepam (N05BA08)	9.3	9.2	8.6	−0.7	−7.2%
ARSALE					
Alprazolam (N05BA12)	52.9	55.8	54.7	1.7	3.3%
Lorazepam (N05BA06)	34.2	36.9	36.2	2.1	6.0%
Diazepam (N05BA01)	20.4	20.8	18.5	−1.9	−9.3%
Ethyl loflazepate (N05BA18)	10.7	7.3	11.4	0.7	6.6%
Bromazepam (N05BA08)	11.0	11.5	10.9	−0.1	−1.2%
ARSALG					
Alprazolam (N05BA12)	37.5	40.3	36.8	−0.7	−1.8%
Lorazepam (N05BA06)	25.6	26.5	1.5	−0.3	−1.0%
Diazepam (N05BA01)	11.4	11.4	10.1	−1.2	−10.9%
Ethyl loflazepate (N05BA18)	9.9	7.0	9.8	−0.1	−0.8%
Bromazepam (N05BA08)	10.7	11.4	10.3	−0.4	−4.0%
ARSLVT					
Alprazolam (N05BA12)	50.4	53.1	56.3	6.0	11.9%
Lorazepam (N05BA06)	36.1	37.6	39.4	3.2	8.9%
Diazepam (N05BA01)	19.6	19.6	19.4	−0.2	−0.8%
Ethyl loflazepate (N05BA18)	13.9	10.2	15.9	2.0	14.2%
Bromazepam (N05BA08)	11.9	12.2	12.7	0.8	6.8%

DID—defined daily dose (DDD) per 1000 older adults per day (DID); ∆—change. Source: Statistics Portugal (Instituto Nacional de Estatística—INE) [21].

## Data Availability

Data were collected from a public database (https://bicsp.min-saude.pt/pt/investigacao/Paginas/medicamentoprescritor_publico.aspx?isdlg=1, accessed on 12 November 2021).

## Supplementary Materials

The following supporting information can be downloaded at: https://www.mdpi.com/article/10.3390/healthcare10071342/s1, Table S1: Excluded benzodiazepines with reasons (n = 20); Table S2: Demographic data of study population in 2019; Table S3: Demographic data of study population in 2020; Table S4: Demographic data of study population in 2021; Table S5: Female and male relation for every BZD, in every ARS, over the last 3 years; Table S6: N05B and N05C national percentage (%) over the last 3 years; Table S7: N05B-TOP5 national percentage (%) over the last 3 years; Table S8: Gender and age group differences in alprazolam, lorazepam, diazepam, ethyl loflazepate, and bromazepam prescription according to each ARS and over the years; Figure S1. A–E: Distribution of ARS in relation to prescription (DID) of (A) alprazolam, (B) lorazepam, (C) diazepam, (D) ethyl loflazepate, and (E) bromazepam; Figure S2: Portugal’s continental national map divided by ARS.
