# Supplementary material for: Trends and Geographic Variabilities in Benzodiazepines Prescription in Primary Care to Older Adults: A 3-Year Population-Based Ecological Study in Portugal
Source: Healthcare (Basel). 2022 Jul 19;10(7):1342. doi: 10.3390/healthcare10071342 (PMC9324858; doi:10.3390/healthcare10071342)
Supplement: Supplementary file 1 [file healthcare-10-01342-s001.zip › Supplementary Material.pdf]

Supplementary Materials: Trends and Geographic Variabilities in Benzodiazepines Prescription in Primary Care to Older Adults: A 3-year Population-based Ecological Study in Portugal

Table S1: Excluded benzodiazepines (N05BA and N05CD groups) with reasons (n=20).

**ATC Code**	**Benzodiazepine**	**Reason of exclusion**
N05BA03	Medazepam	(a) BZD without Marketing Authorization (MA) or had lost MA in Portugal during the study period
N05BA07	Adinazolam	
N05BA13	Halazepam	
N05BA14	Pinazepam	
N05BA15	Camazepam	
N05BA16	Nordazepam	
N05BA17	Fludiazepam	
N05BA19	Etizolam	
N05BA21	Clotiazepam	
N05BA23	Tofizopam	
N05BA24	Bentazepam	
N05CD02	Nitrazepam	
N05CD03	Flunitrazepam	
N05CD06	Lormetazepam	
N05CD10	Quazepam	
N05CD12	Doxefazepam	
N05CD13	Cinolazepam	
N05CD15	Nimetazepam	
N05BA10	Ketazolam	(b) without a defined daily dose, accordingly to the SIM@SNS online platform and to the ATC/WHO Index
N05CD14	Remimazolam	(c) exclusively used in a hospital setting

Table S2: Demographic data of study population in 2019.

ARS/Sex/Age group	Male		Female		Total Male	Total Female	Total
	65-74 y	≥ 75 y	65-74 y	≥ 75 y			
ARSN	192926	144787	233886	233514	337713	467400	**805113**
ARSC	97257	86499	116017	139950	183756	255967	**439723**
ARSLVT	179604	146041	225182	232645	325645	457827	**783472**
ARSALE	25946	25437	29830	41220	51383	71050	**122433**
ARSALG	22356	19261	25718	28718	41617	54436	**96053**
**Total**	**518089**	**422025**	**630633**	**676047**	**940114**	**1306680**	**2246794**

Table S3: Demographic data of study population in 2020.

ARS/Sex/Age group	Male		Female		Total Male	Total Female	Total
	65-74 y	≥ 75 y	65-74 y	≥ 75 y			
ARSN	184555	136395	222341	217495	320950	439836	**760786**
ARSC	101226	89265	120469	145101	190491	265570	**456061**
ARSLVT	179663	149744	225566	237389	329407	462955	**792362**
ARSALE	26182	25023	30011	40659	51205	70670	**121875**
ARSALG	22488	19431	26097	28990	41919	55087	**97006**
**Total**	**514114**	**419858**	**624484**	**669634**	**933972**	**1294118**	**2228090**

Table S4: Demographic data of study population in 2021.

ARS/Sex/Age group	Male	Female	Total
	≥ 65 y	≥ 65 y	
ARSN	348774	459350	**808124**
ARSC	216461	284064	**500525**
ARSLVT	328745	456804	**785549**
ARSALE	56085	73681	**129766**
ARSALG	50932	60048	**110980**
**Total**	**1000997**	**1333947**	**2334944**

Table S5: Female and Male Relation for every BZD, in every ARS, over the last 3 years (Intermediate Calculations).

	Mulheres					***Homens***				
	**Alentejo**	**Algarve**	**Norte**	**Centro**	**LVT**	**Alentejo**	**Algarve**	**Norte**	**Centro**	**LVT**
Midazolam	522680	226713	1083398	561606	1552419	182709	118774	532216	238398	646178
Temazepam	82936	55916	201684	60648	456191	24836	18760	56154	21462	123438
Triazolam	198740	136580	848760	240180	1079220	57260	60740	342140	102040	307400
Estazolam	484111	195049	1971770	793193	2820675	157901	81229	866068	318659	1028151
Flurazepam	189340	76893	1103591	304569	1033614	71828	45497	476762	138764	419926
Cloxazolam	405190	164735	3578155	2017650	2391650	153930	65305	1464715	683345	908230
Ethyl Loflazepate	1021940	760320	10224400	4282520	8619940	321660	233580	3828640	1457940	2870320
Alprazolam	5704255	3016795	43277505	29277115	33944515	1738865	1219150	16509195	10427580	12011745
Clobazam	122410	83695	1731785	544555	985195	49875	27535	795575	250490	414085
Bromazepam	1156890	870312	8008239	3452202	8044782	366417	325449	2619726	1150407	2551647
Potassium Clozepate	322690	156310	2124440	1368855	1754525	90720	61335	720745	445465	585185
Chlordiazepoxide	2760	950	3993	633	2303	1000	290	2303	403	1163
Diazepam	1927097	816383	11813788	8685364	12176502	793779	287032	4841784	3258322	4666546
Prazepam	520	1880	23773	3313	5920	347	573	8553	1520	1140
Oxazepam	98034	90060	1077177	1541994	688461	69126	70671	1606068	1341534	527391
Lorazepam	3642170	2060188	47619206	23890720	23928480	1245136	805362	19788884	9189372	8591362
Cetazolam	0	0	0	0	0	0	0	0	0	0
Brotizolam	574728	273756	2462866	642292	2457266	197232	98812	1903930	254940	936922
Loprazolam	331080	74430	1133280	304635	826725	112485	37740	518580	143205	309780
Mexazolam	2431	2183	10415	8549	13707	766	729	3651	2715	4529
TOTAL	16790002	9063148	1.38E+08	77980593	102782090	5635872	3558563	56885689	29426561	36905138

Table S6: N05B and N05C National Percentage (%), over the last 3 years.

**Grupos ATC 3rd level**	**N05C**	**N05B**
Total 3 anos	35477073	441125565
%	7.4%	92.6%

Table S7: N05B-Top5 National Percentage (%), over the last 3 years.

	**Alprazolam**	**Lorazepam**	**Diazepam**	**Ethyl Loflazepate**	**Bromazepam**	**TOTAL**
**Top 5**	**N05BA12**	**N05BA06**	**N05BA01**	**N05BA18**	**N05BA08**	
**Total**	157126720	140760880	49378727	33621260	28546071	
**% do total**	33.0%	29.5%	10.4%	7.1%	6.0%	85.9%

Table S8: Gender and Age Group differences in Alprazolam, Lorazepam, Diazepam, Ethyl Loflazepate and Bromazepam prescription according to each ARS and over the years.

DDD/ 1000 inhabitants[a]/day (DID)		2019				2020				2021*		Δ 2019-2021	
		Female		*Male*		*Female*		*Male*		*Female*	*Male*	*Female*	*Male*
		**65-74**	**≥ 75**	**65-74**	**≥ 75**	**65-74**	**≥ 75**	**65-74**	**≥ 75**				
**ARSN**	**Alprazolam**	83.7	73.7	41.1	41.8	94.3	85.4	46.2	49.2	91.8	45.8	86.7	44.9
	**Lorazepam**	75.1	99.6	42.9	59.1	84.7	113.3	48.4	67.0	100.3	55.2	95.4	53.8
	**Diazepam**	24.2	20.0	12.9	12.0	27.1	22.3	14.4	13.5	24.2	13.0	23.7	13.2
	**Ethyl Loflazepate**	25.0	17.2	12.2	9.6	19.7	13.6	9.8	7.8	23.5	11.1	20.5	10.4
	**Bromazepam**	14.2	15.8	6.2	7.8	15.4	17.9	6.5	8.8	16.5	7.0	16.0	7.1
**ARSC**	**Alprazolam**	98.1	101.4	45.7	53.7	98.3	102.5	45.5	55.4	98.0	45.7	99.5	48.3
	**Lorazepam**	68.0	91.6	34.7	52.5	68.2	92.8	36.3	52.9	81.0	40.9	81.2	42.6
	**Diazepam**	34.1	28.1	16.2	15.6	32.6	28.2	16.1	15.5	27.7	13.8	29.5	15.1
	**Ethyl Loflazepate**	19.8	13.9	8.6	7.0	13.4	9.3	6.1	4.7	15.9	5.0	14.6	6.8
	**Bromazepam**	10.2	13.4	4.5	6.7	10.0	13.5	4.4	6.9	11.4	7.0	11.7	5.3
**ARSLVT**	**Alprazolam**	61.2	66.3	29.0	34.4	64.6	69.0	31.3	36.9	71.7	34.9	67.5	33.4
	**Lorazepam**	34.0	56.9	17.0	29.8	35.5	58.3	18.4	31.2	49.8	24.8	47.6	23.9
	**Diazepam**	24.9	23.7	12.8	13.3	24.9	23.6	12.8	13.4	24.1	12.9	24.2	13.0
	**Ethyl Loflazepate**	20.1	15.7	9.0	7.6	15.0	11.1	6.6	5.9	20.6	9.4	17.1	8.0
	**Bromazepam**	13.6	17.4	5.8	8.2	13.6	17.8	6.1	8.4	16.7	7.3	16.0	7.1
**ARSALE**	**Alprazolam**	68.9	70.8	25.5	33.0	72.8	75.2	27.4	33.9	73.3	30.2	72.5	30.0
	**Lorazepam**	34.2	51.6	15.1	25.2	36.5	55.1	17.3	28.2	47.3	21.6	49.0	22.8
	**Diazepam**	26.2	24.5	13.8	14.0	27.3	24.1	14.7	14.2	22.9	12.8	24.5	13.7
	**Ethyl Loflazepate**	17.4	11.7	6.3	5.9	12.1	7.5	4.2	4.3	15.4	6.3	13.0	5.6
	**Bromazepam**	13.5	15.1	5.3	7.5	14.2	15.7	5.5	7.8	14.6	6.0	14.7	6.3
**ARSALG**	**Alprazolam**	47.2	48.0	23.6	25.1	51.2	49.8	25.8	28.3	48.1	23.5	48.7	24.8
	**Lorazepam**	24.5	40.1	13.0	20.3	25.7	40.5	14.9	20.0	33.6	15.7	33.3	16.4
	**Diazepam**	14.8	12.9	8.2	8.2	14.5	12.5	8.4	9.2	12.4	7.5	13.2	8.1
	**Ethyl Loflazepate**	15.5	11.7	5.5	4.9	11.0	7.7	4.5	3.6	13.9	5.0	12.3	4.8
	**Bromazepam**	12.2	15.2	5.5	8.0	13.2	15.8	6.3	8.5	13.8	6.1	14.1	6.6

* DID stratified by age was calculated only between 2019 and 2020 since official data for the older population in the year of 2021 are grouped only in "over 65 years".

Figure S1 A-E: Distribution of ARS in relation to prescription (DID) of (A) Alprazolam, (B) Lorazepam, (C) Diazepam, (D) Ethyl Loflazepate and (E) Bromazepam.

A

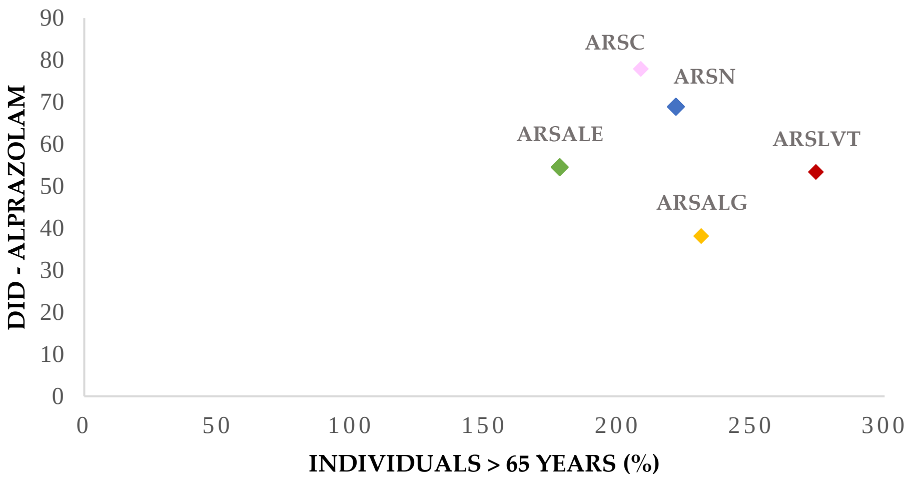

B

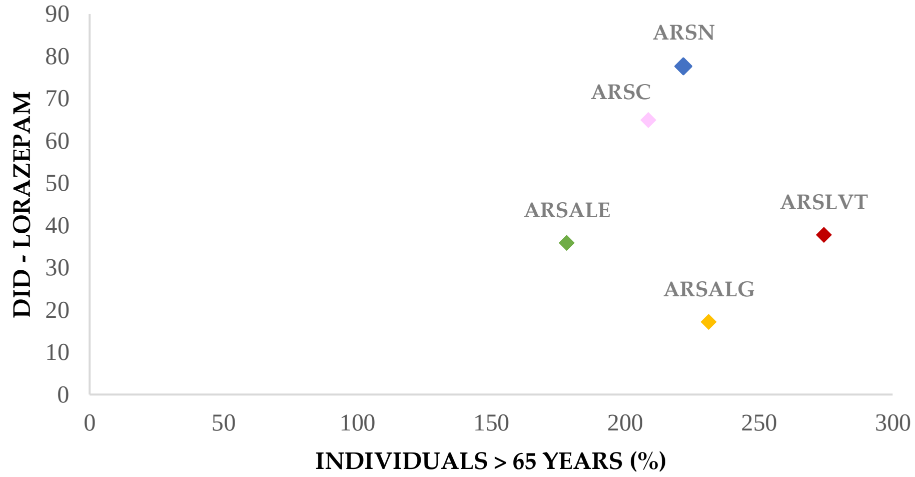

C

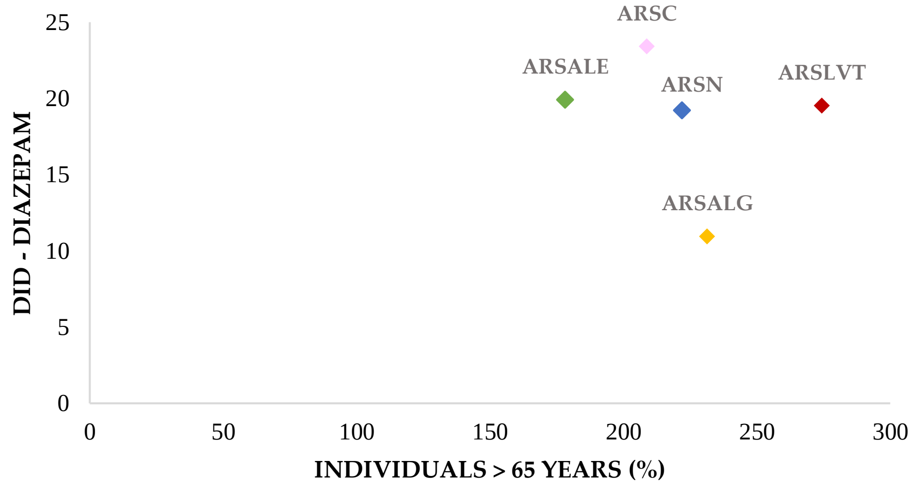

D

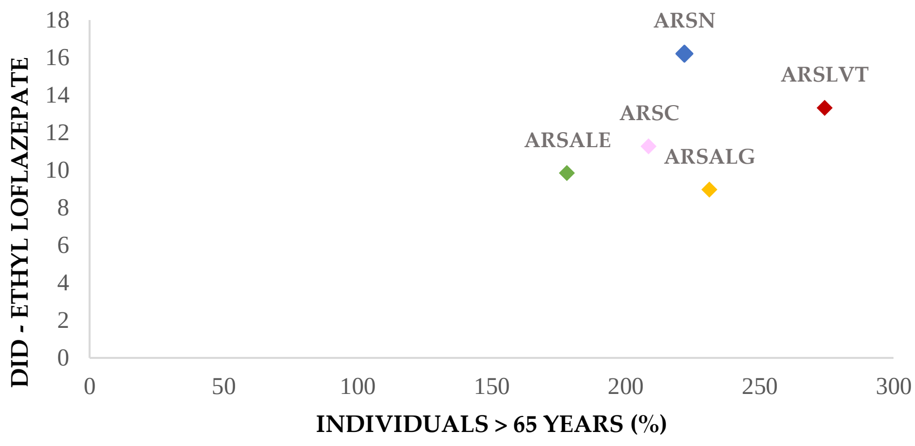

ARSN
ARSLVT
ARSC
ARSALE
ARSALG
DID - ETHYL LOFLAZEPATE
18
16
14
12
10
8
6
4
2
0
0
50
100
150
200
250
300
INDIVIDUALS > 65 YEARS (%)

E

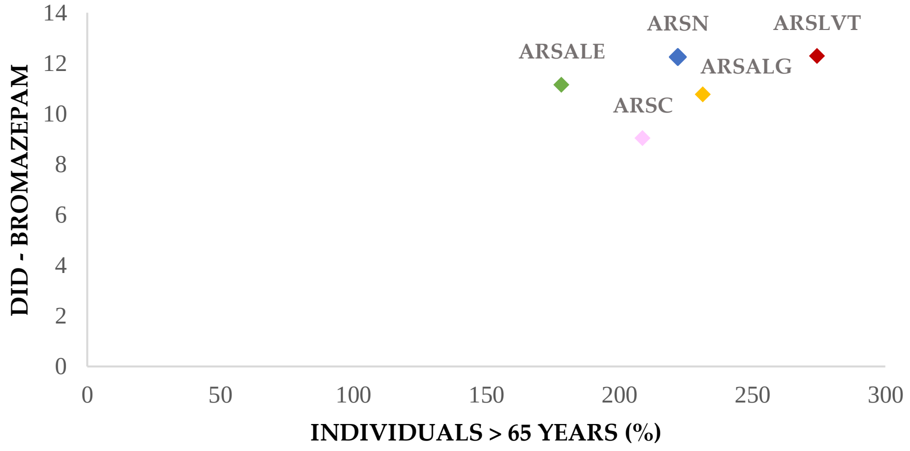

ARSN
ARSLVT
ARSALE
ARSALG
ARSC
DID - BROMAZEPAM
14
12
10
8
6
4
2
0
0
50
100
150
200
250
300
INDIVIDUALS > 65 YEARS (%)

Figure S2: Portugal's Continental National Map divided by ARS

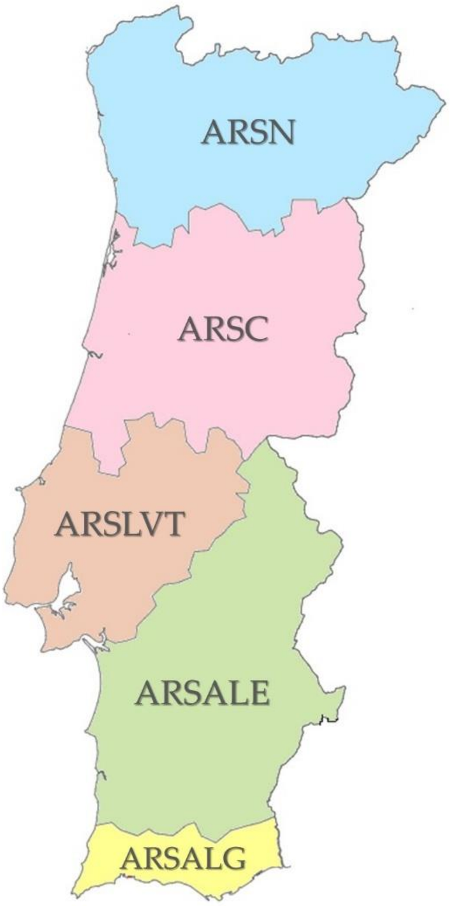

Legend:

ARSN – North Regional Health Administration (*Administração Regional de Saúde do Norte*)
ARSC – Center Regional Health Administration (*Administração Regional de Saúde do Centro*)
ARSLVT – Lisbon and Tagus Valley Regional Health Administration (*Administração Regional de Saúde de Lisboa e Vale do Tejo*)
ARSALE – Alentejo Regional Health Administration (*Administração Regional de Saúde do Alentejo*)
ARSALG – Algarve Regional Health Administration (*Administração Regional de Saúde do Algarve*)
